# Information-based cues at point of choice to change selection and consumption of food, alcohol and tobacco products: a systematic review

**DOI:** 10.1186/s12889-018-5280-5

**Published:** 2018-03-27

**Authors:** Patrice Carter, Giacomo Bignardi, ​Gareth J. Hollands, Theresa M. Marteau

**Affiliations:** 1 Behaviour and Health Research Unit, University of Cambridge, Cambridge, UK; 2 Centre for Outcomes Research and Effectiveness, Research Department of Clinical, Educational and Health Psychology, University College London, 1-19 Torrington Place, London, WC1E 7HB UK

**Keywords:** Food, Alcohol, Information-based cues, Point of choice, Selection, Consumption

## Abstract

**Background:**

Reducing harmful consumption of food, alcohol, and tobacco products would prevent many cancers, diabetes and cardiovascular disease. Placing information-based cues in the environments in which we select and consume these products has the potential to contribute to changing these behaviours.

**Methods:**

In this review, information-based cues are defined as those which comprise any combination of words, symbols, numbers or pictures that convey information about a product or its use. We specifically exclude cues which are located on the products themselves. We conducted a systematic review of randomised, cluster- randomised, and non-randomised controlled trials to assess the impact of such cues on selection and consumption. Thirteen studies met the inclusion criteria, of which 12 targeted food (most commonly fruit and vegetables), one targeted alcohol sales, and none targeted tobacco products.

**Results:**

Ten studies reported statistically significant effects on some or all of the targeted products, although studies were insufficiently homogenous to justify meta-analysis. Existing evidence suggests information-based cues can influence selection and consumption of food and alcohol products, although significant uncertainty remains.

**Conclusions:**

The current evidence base is limited both in quality and quantity, with relatively few, heterogeneous studies at unclear or high risk of bias. Additional, more rigorously conducted studies are warranted to better estimate the potential for these interventions to change selection and consumption of food, alcohol and tobacco products.

**Trial registration:**

*PROSPERO*. 2016;CRD42016051884.

**Electronic supplementary material:**

The online version of this article (10.1186/s12889-018-5280-5) contains supplementary material, which is available to authorized users.

## Background

Non-communicable diseases, including cardiovascular disease, many cancers, diabetes, and chronic respiratory disease are the leading cause of death worldwide [1]. Importantly, major risk factors for these diseases are modifiable health behaviours, including smoking, high alcohol intake, excessive consumption of food and physical inactivity [2] These behaviours are multifaceted and driven by numerous factors, including cues in the environments that surround us, often without our awareness [3–5]. Altering cues in small-scale environments, or proximal physical micro-environments [6, 7], where people select products, such as grocery stores, restaurants and bars offer an opportunity to influence selection [8] across diverse consumer populations [9]. These include simple information-based cues, which when present within the same environment as that in which the behaviour is performed, and therefore proximal to that behaviour, both spatially and temporally, could influence selection of a target product without the need for extensive or conscious engagement with the information provided.

These information-based cues are different to traditional on-pack nutritional labels, which the EU Food Information Regulations made mandatory for most pre-packaged foods (Regulation (EU) 1169/2011) [10]. These regulations are to ensure people have appropriate information to make informed selections about the food they buy and eat. The UK Department of Health has also pledged to increase people’s awareness and understanding of alcohol units, committing alcohol producers to label their products with unit and health information [11]. While these regulations are supported by evidence that labelling can be an effective means of helping consumers choose healthier products [12, 13], labels are not consistently read and used by consumers [14, 15]. A recent review estimated that among students and young adults, only about 37% used labels [16]. It has also been proposed that nutritional information on labels, which may contain detailed information on the quantities of a range of nutrients, often in multiple formats on the same product, is too complex to be readily understood and used by some populations [17].

Given these considerations, the implementation of simple information-based environmental cues which are placed within the micro-environment, but not directly on the product, represent a complementary means of influencing people’s behaviour. Such information-based cues, sometimes also referred to as point of choice (POC) or point of purchase (POP) interventions may have the potential to influence selection of products across large proportions of the population, including those who do not readily engage with more detailed, on-product labelling [18]. Previous data of on-product labelling exist [12, 13], however, to our knowledge, there has been no attempt to systematically review their potential impact of information-based cues placed within the physical environment. The aim of the current review is to estimate the effect of information-based cues on selection and consumption of food, alcohol and tobacco products.

## Methods

Following the PRISMA guidelines [19] and the Cochrane Handbook for Systematic Reviews [20] we developed a protocol which was registered on the PROSPERO international Prospective Register of Systematic Reviews database in advance of the review being conducted [21].

### Criteria for inclusion in the review

### Types of studies

To be included in the review studies were required to be randomised controlled trials, or cluster-randomised trials. Non-randomised controlled trials were only included when investigators had attempted to standardise the groups and minimise allocation bias [22].

### Types of interventions

Interventions considered eligible for this review were those which involved the comparison of the effect of an information-based cue at point of choice on food, alcohol or tobacco selection or consumption to that of a non-information-based cue condition. Interventions included those conducted in any out-of-home environment, where an individual had a range of food, alcohol or tobacco products to select from; including grocery stores, supermarkets, restaurants, bars, school canteens, and workplace cafeterias. Eligible studies were required to report unregulated selection or consumption (with or without purchasing) of food, alcohol or tobacco item(s). Unregulated refers to the behaviour of individuals not being regulated by explicit instructions or actions of the researcher.

### Definition of information-based-cue

Information-based cues were defined as those comprising any combination of words, symbols, numbers or pictures that convey information about the product or its use (including the impact of its use) [7]. The media by which the information-based cues were communicated could include: point of purchase advertising boards, display stands, banners, posters, flyers, and labelling on store equipment (e.g. shelving, shopping trolley, baskets, cafeteria trays). Standardised in-store announcements (e.g. those conveyed over a speaker system) and standardised information on screens within the out-of-home environment were also included.

### Exclusion criteria

Interventions were excluded if the information-based cue was placed directly on the product of interest (i.e. the consumable substance and its immediate or integral packaging), for examples studies which investigated the effect of traditional food labels (back of pack and front of pack labels), or interventions which provided warning messages on tobacco products (noting that systematic reviews are available on nutritional labelling [12, 13, 23]. and tobacco warning labels [24]). Information-based cues which provided specific nutrient or energy claims, (e.g. “low-fat”), those which provided the nutritional content of a product, and those which listed specific nutritional values (e.g. amount of fat, carbohydrate, sugar, salt or energy content, alcohol volumes) were excluded. Information-based cues provided through the Internet or television, interventions targeted or tailored to specific individuals, or those that were designed to be delivered in an interactive fashion (for example interventions delivered in real-time by humans) were excluded. Studies involving non-human participants, and those for which the information-based cue concerned food allergens (e.g. gluten free information) were excluded. No other exclusion criteria were set.

### Search strategy

To ensure a complete yet precise search was conducted, we developed a strategy to include medical subject headings and free text terms based on the eligibility criteria. The search was initially developed for MEDLINE ((OvidSP In-Process 1946 to 25th November 2016) (Additional file 1 Table S1), and tested for sensitivity to retrieve a set of reference papers. The search strategy was then adapted for EMBASE (OvidSP 1974 to 26th November 2016), PsycINFO (OvidSP inception to 26th November 2016), Cochrane Central Register of Controlled Trials (CENTRAL 1992 to 26th November 2016) and Web of Science (inception to 25th November 2016), using each individual database thesauri and notes. There was no restriction on publication date, format or language. Reference lists of all eligible articles were searched, and we additionally conducted forward citation tracking using Google Scholar.

### Study selection

All abstracts were imported into a reference manager software package to facilitate selection, duplicates were removed and abstracts screened against the eligibility criteria by the lead author. Potentially relevant full texts were obtained and screened independently by two authors. Disagreements were resolved via discussion and a third author acted as arbiter where necessary.

### Risk of bias

All articles were assessed using the Cochrane Collaboration risk of bias tool [20]. The tool comprises six specific domains of potential bias: selection bias, performance bias, detection bias, attrition bias, reporting bias and any other sources of bias. The tool was applied to each included study individually by two authors and justification for each judgement of bias (low, unclear or high risk) for each domain was recorded. Any disagreements were resolved by discussion by the two authors, with a third author acting as arbiter where required. Following these guidelines [20] an overall summary ‘Risk of bias’ judgement (low, unclear or high risk) for each study was derived based on the included domains. This means an article was only considered to have low risk of bias if all domains were judged as low risk. If any one domain was judged as high risk, the overall summary was judged as high risk of bias.

### Study synthesis

Due to the heterogeneous nature of the included articles in terms of intervention, study design and participant characteristics, the results were synthesised narratively. The included articles reported outcome data in various ways; therefore, we have not attempted to standardise outcomes, but present data as stated by authors in each included article.

## Results

**Fig. 1:**
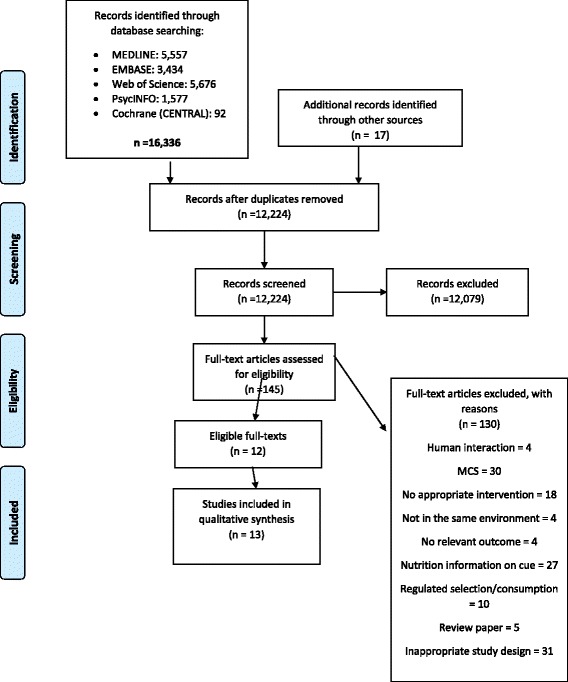
PRSIMA flow diagram

The search strategy generated a total of 12,224 potentially eligible articles, after removing duplicates. Abstract and title screening identified 145 full-text articles that had the potential to be included. Twelve articles (which included 13 studies) met all inclusion criteria, and were included in the review. Details, including reasons for exclusion are shown in the PRISMA flow diagram (Fig. 1).

### Included studies

Seven studies were conducted in the United States of America [25–31], three in the Netherlands [32–34], two in Switerland [35] and one in France [36]. Three of the studies targeted selection by children [26, 31, 35], one targeted purchases by university students [28], three studies were conducted in the workplace [32, 33, 35] and one in bars [34]. The remaining studies were conducted in locations used by both adults and children [25, 27, 29, 30], although the most likely consumers would be adults.

### Product of interest

Twelve of the 13 studies targeted food intake, eight studies included a measure of fruit and vegetable selection or consumption, of which six solely targeted fruit and vegetables [25, 26, 29–31, 36]. Four of the studies aimed to increase the selection of healthier food and beverage items or reduce selection of unhealthy options [27, 28, 32, 33]. Two studies targeted selection of both healthy and unhealthy snacks [35]. One study targeted alcohol sales [34], and we did not identify any studies targeting tobacco products.

### Characteristics of the information-based cue

#### Medium of cue

Of the 13 included studies, 11 examined the effect of a visual information-based cue. The primary medium across the different studies included signs [25, 32, 36], vinyl banners [26], TV segments [26], menu boards [27], posters [35], placards [28, 33], and arrows placed on the floor [30]. A number of the studies included secondary information-based cues, for example, additional posters [27, 28, 32], paper menus [27], and table tents [28, 32]. The remaining two studies investigated the use of audio information-based cues, one of which examined the effect of a morning audio announcement in schools [31] and the other investigated music being played in bars [34].

#### Content of the cue

Of the 11 studies that examined the effect of a visual information-based cues, five studies included bright colours with images of the product of interest [25–30], for example images of fruit and vegetables, as the content of their cue. Two studies included posters which depicted different images, including Giacometti sculptures, a nature scene, an activity scene or a fun fair [35]. Three studies incorporated a logo [27, 32, 33] and one study had simple writing on a white easel [36]. Of the two audio information cues, one included messages featuring a magical superhero, “bean man” [31] and the other played contemporary popular music which included lyrics with reference to alcohol [34].

#### Location of the intervention

Six of the studies were conducted in cafeterias, three of which were school based cafeterias [26, 31, 36], two were worksite cafeterias [32, 33], and one university dining halls [28]. Three studies were based in supermarkets [25, 29, 30], one in take-away restaurants [27], one in bars [34], one was in university buildings [35] and one in workplace buildings [35].

#### Outcome measures

The outcome measures varied across studies. Data were not presented in full for many of the included studies. Five studies measured sales data [25, 27, 32–34]. Other reported outcomes included proportion of produce spending to total food spending per person per day [29, 30], percentage snack choice [ [35], frequency of choice [31, 36], total number and percentage of children selecting vegetables [26], and reported intake of fat, fibre and vegetables [32, 33].

#### Risk of bias

**Fig. 2:**
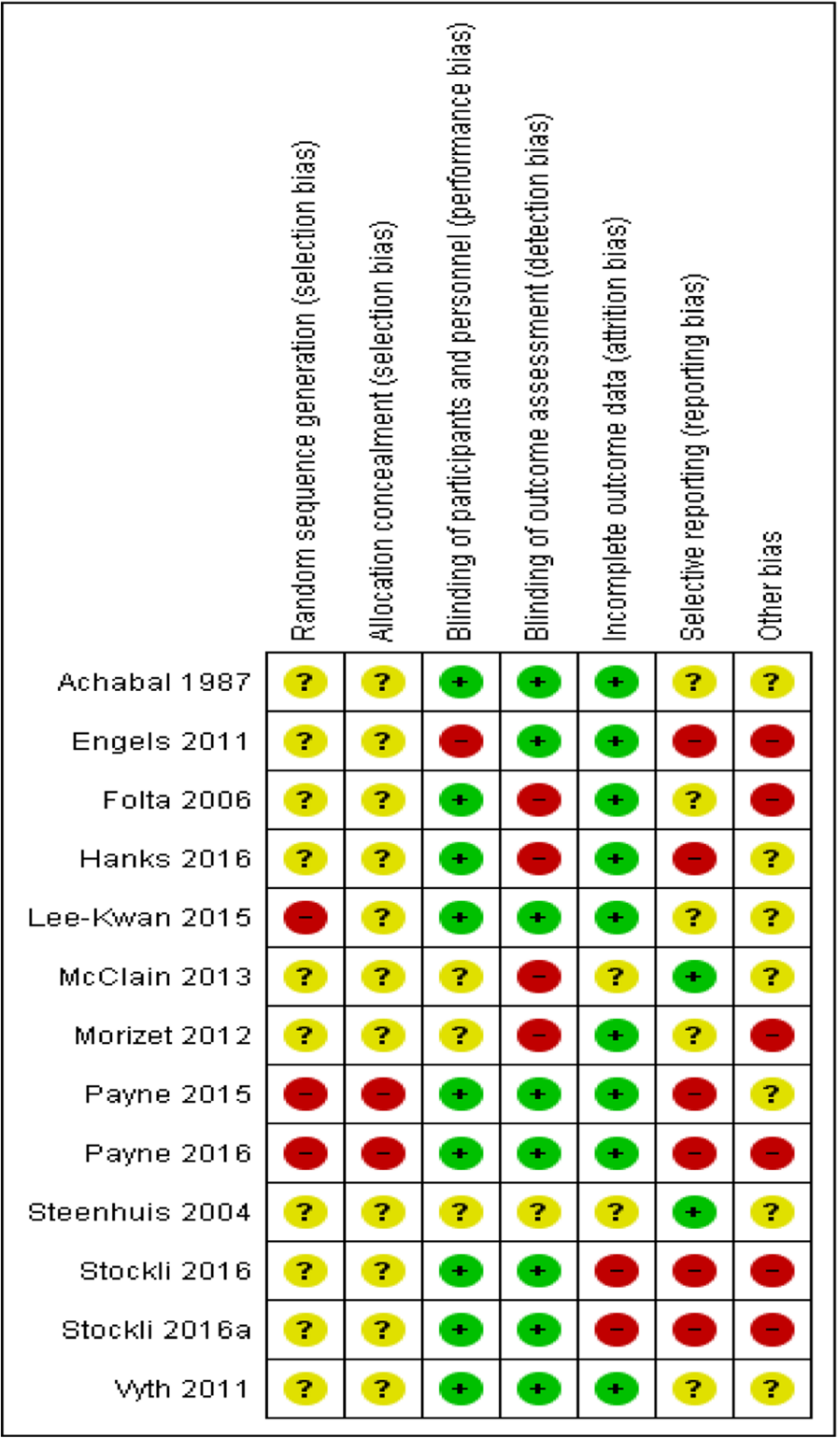
Risk of bias summary figure for included studies. Stockli 2016 and stockli 2016a refer to study 1 and study 2 respectively, both from Stockli et al 2016 [35]

**Fig. 3:**
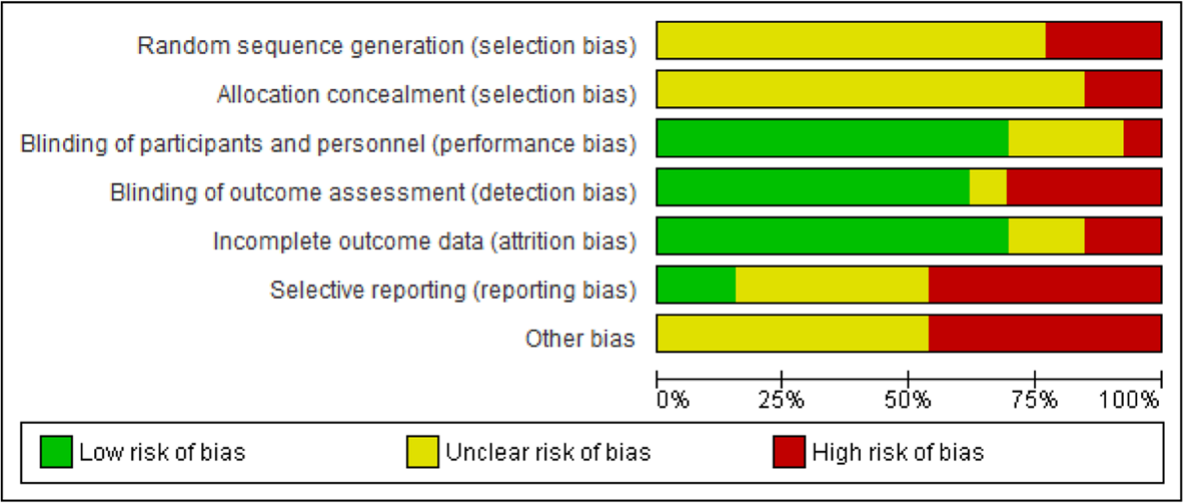
Summary risk of bias graph

For the overall summary of bias, three studies were judged to have unclear risk of bias [25, 32, 33] and ten had a high risk of bias [26–31, 34–36]. Notably, all studies had either unclear or high risk of bias in relation to the method of random sequence generation. Only two studies had low risk of bias for the domain of selective reporting [28, 32]. Figure 2 provides judgements for each domain across each included study and Fig. 3 summarises risk of bias judgements across included studies. Full details of review authors’ judgements are presented in Additional file 2 Table S2.

#### Intervention effect

Ten studies reported a statistically significant intervention effect on some or all products. Three observed no evidence for an intervention effect within their primary analysis. Although results were mixed, the direction of findings was generally in favour of the intervention increasing selection of healthier products or decreasing selection of unhealthy products. Full details are presented in Table 1.

**Table 1:** Results of the included interventions

Author, reference and country	Design	Study setting	Product and cue category	Information-based cue intervention			Comparison	Duration	Outcome measures	Results	Result
				Medium of cue	Content of cue	Location of cue					
Cues concerning attention											
Achabal 1987 [25] USA	Randomised controlled trial	Supermarket	Fruit and vegetables	Colour signs	Pictures of produce (broccoli, cabbage, carrots, cauliflower, kiwi, tomatoes), plus purchase selection information	Signs placed in a holder strip six feet off the floor, directly about the produce	No signs in store Additional arm: Nutrition signs, which included the same information as the intervention arm, plus calorie content and a panel of key nutrients.	4 weeks (plus 4 weeks pre-test, and 4 weeks post-test)	Weekly sales data	No variation in sales were observed by the presence of the sign, with no significant effect of the sign (F = 0.684, *p* = < 0.505). Additional analysis comparing matched store data, found no differences in the number of times the sales were greater in the intervention stores than the control stores.	–
Payne 2015 [29] USA	Pilot study	Supermarket	Fruit and vegetables	Brightly coloured placards	Graphics of popular fruit and vegetables, plus positive and social norm messages	Placards were placed on the inside front and outside front of the grocery cart	No intervention	14 days (plus 57 days matched pre-intervention period)	Weekly sales data	There was an increase in average spending per day per person on produce of 16% (< 0.01) in the intervention store and an increase of 4% (*p* = n.s.) in the control store.	+
Payne 2016 [30] USA	Pilot study	Supermarket	Fruit and vegetables	Large green arrows	Green arrows included graphics of fruits and vegetables, an emoticon to facilitate social approval and text such as “follow green arrow for health”	Arrows were placed around the store perimeter, on the floor in highly visible places	No intervention	14 days (plus 14 days pre-intervention period)	Weekly sales data	There was an increase on produce spending per day per person of 8% (*p* = 0.01). The authors do not state change in spending in the control store.	+
Hanks 2016 [26] USA	Randomised controlled trial	School cafeterias	Fruit and vegetables	Brightly coloured vinyl banners and/or TV segments	Branded media vegetable characters with human characteristics and super human strength.	Banners were placed around the salad bar and on top of the bar. TVs were placed on small tables near the salad bar Small printed vegetables were also placed on the floor to direct children to the salad bar	No Intervention	4 weeks (plus 2 weeks pre-intervention)	Food preperation records and count data of children serving themselves	There was an increase from 60 to 185 daily vegetable servings in the schools with the combined vinyl banner and TV segments intervention. There was a reported increase in servings for the individual intervention arms, but data were not significant. Count data showed a significant increase in frequency of children taking vegetables from the salad bar; 12.6 to 24% with vinyl banners, 10.2% to 34.6% with vinyl banners and TV segments. There was no significant change with just the TV segments (13.8% to 18.9%) or in the control schools.	+/−
Morizet 2012 [36] France	Experimental design (randomised by temporal order and condition)	School cafeterias	Fruit and vegetables	A white paper easel with text information	A basic label, “new carrot/broccoli recipe” and a model label: “new carrot/broccoli recipe, special mix for superheroes”	Labels were presented at the front of the gastronorm tray where vegetables were located.	No label	Two different days (one per vegetable option)	Choice frequency of vegetables	Children selected the familiar carrot or broccoli dish significantly more often when no label was present. For the carrot dish there was a significant difference in the number of children who selected the novel dish with the basic label (*p* = 0.012), and the model label (*p* = 0.002) as compared to no label condition. The patterns were similar for broccoli, but not statistically significant.	+/−
Folta 2006 [31] USA	Randomised controlled trial	School cafeterias	Fruit and vegetables	Audio announcement	Messages promoted beans and featured a magical superhero “bean man”	Audio messages were played during the school morning announcements	The same bean dishes were introduced to the school lunch menus. The children received their normal morning announcements, without the addition of any health messages	Approximately 3 months	Choice of beans	Overall data showed no difference in frequency of bean selection between intervention and control schools.	–
Cues providing educational information about the product properties											
Steenhuis 2004 [32] Netherlands	Randomised pre-test, post-test experimental design	Worksite cafeterias	Low fat options	Signs (plus brochures, table tents and optional self-help manuals, newsletters and badges (the additional factors were considered as part of the educational programme)).	The signs consisted of the program logo, the name of the food item, and an indication that it was a healthy choice	Signs were placed in front of targeted food products	No intervention Additional arms: Educational programme, Food supply plus education.	One month (with the option to extend to six months)	Sales proportion of low-fat products per product group A self-administered food frequency questionnaire to measure fat, fruit and vegetable intake.	At one and six months there were no significant changes in reported fat, fruit or vegetable consumption. Sales data showed a significant increase in the proportion of low fat desserts sold in the intervention sites as compared to control sites. No other differences in sales were observed.	+/−
Vyth 2011 [33] Netherlands	Cluster randomised controlled trial	Worksite cafeterias	Low fat options plus fruit	Placards with a logo. Menus explaining the logo were available.	The logo consisted of a tick, indicating a healthy choice. The logo was part of “The choices intervention” which assigns its logo to foods which meet a determined sodium, added sugar, saturated fat, trans fats, fibre and energy.	The placards were placed next to freshly prepared “choices” sandwiches and soups, and the fruit basket.	No labelling or any other communication regarding the intervention	3 weeks (plus 3 weeks pre and 3 weeks post intervention periods)	Daily sales data	There was a significant difference in fruit sales between the intervention and control sites during the intervention period. No other differences in sales of targeted products were observed.	+/−
McClain 2013 [28]USA	Cluster randomised controlled trial with repeated cross sectional assessment	University dining halls	Low fat options	Placards, posters and table tents	Colourful photographs, and messages such as “brain food”	Placards were placed at food stations and on dining tables. Posters were placed around the dining halls.	No intervention	4 weeks (plus 7 days pre and post intervention)	Harvard food frequency questionnaire data	Students in the control halls consumed significantly more junk food and high fat meat products from baseline to intervention relative to students in the intervention halls. No other differences were observed.	+/−
Lee-Kwan 2015 [27] USA	Quasi-experimental study	Carryout restaurants	Healthy entrees, sides and beverages	Menu boards and posters Paper menus were also available, which were replicates of the menu boards	Digital colour photographs of selected healthier options plus a green leaf logo to indicate the healthier options	Menu boards were placed next to healthier items. Posters were placed next to the menu boards.	No intervention	8 weeks (plus 4 week pre-intervention, and a further 16 week intervention of additional interventions)	Weekly sales data	In the intervention group the relative odds of healthy entrees were greater during the intervention period to baseline (OR 1.16, 95%CI: 1.08: 1.26). The changes were not significantly different to control sites.The relative odds of healthy sides and beverages was not significantly different over time in the intervention group. were no significant differences in units sold from baseline in the intervention group or compared to control.	–
Implicit cues											
Stockli 2016 [35] Switzerland	One-factorial within subjects experimental design study	Vending machines in University and Public Health office buildings	Both healthy and unhealthy snacks	Posters	Study 1: A nature poster, (showing grassland, tress and a blue sky with clouds) an activity poster (showing running legs in sports shoes and asphalt in the background) and a fun fair poster (showed two carousels with a summery blue sky in the background) Study 2: A poster with the Giacometi sculptures, (elongated figures) an activity poster and a fun fair poster (as above)	Posters were placed above vending machines	No posters	4 weeks	Daily sales data	There was a significant association between poster exposure and snack choice in both studies: Study 1: The percentage of healthy snacks selected was, 34% with the nature poster, 28% with the activity poster, 22% with the control condition and 18% with the funfair poster. Study 2: The percentage of healthy snacks selected was 58% with the Giacometti poster, 44% with the activity poster, 29% with the fun fair poster and 21% with the control condition	+
Engles 2011 [34] Netherlands	Experimental design study (evenings were randomised between arms)	Bars	Alcohol	Audio: Musical playlists	A playlist of 90 songs (with a 5.5 h duration) with contextual references to alcohol. Songs contained lyrics that referred to alcohol in them, for example “red, red, wine”.	The music was played on a fixed day of the week, at a fixed starting time and were played in a random order by the bartenders	A playlist of songs created by the same artists included in the intervention playlist, but the songs did not refer to alcohol. To ensure a good match, attention was played to timeframe and period of the album, tempo and energetic content of the songs.	The three included bars collected data on 18, (bar A) 12, (bar B) and 16 evenings (bar c).	Sales of alcoholic drinks corresponding to the two hours the music was played	Turnover was significantly higher on nights when music had reference to alcohol played compared to nights when the control playlist was played (*p* = < 0.05)	+

+ Reported significant increase in healthier items (including fruit and vegetables) or decrease in unhealthier items for primary data. +/− Mixed results for reported increase in healthier items (including fruit and vegetables) or decrease in unhealthier items for primary data. – No evidence of effect on increasing healthier items (including fruit and vegetables) or decreasing unhealthier items for primary data

#### Fruit and vegetables

Two interventions conducted in supermarkets found significant intervention effects on selection of fruit and vegetables. Stores which placed placards on grocery carts observed an increase in average spending on produce per day by 16%, as compared to an increase of 4% in control stores [29]. The placement of green arrows around store perimeters increased average spending on produce per day by 8% compared to baseline (data for the control stores was not provided) [30]. In contrast, a further study conducted in supermarkets found no difference in sales when large signs were placed above produce items [25]. The remaining studies were conducted in cafeterias. One study placed brightly coloured banners around salad bars and played TV segments which included branded vegetable characters, daily servings of vegetables increased from 60 to 185 (*p* = 0.028) over time [26]. A study in France found the presence of labels stating “new recipe” or “special mix for superhero’s” resulted in significantly more children selecting novel vegetables as compared to when no cue was present (no label versus new recipe *p* = 0.012 and no label versus special mix for superhero’s label, *p* = 0.002) [36]. A worksite cafeteria study with a logo based information-based cue found statistically significant effects on the selection of fruit; however, this was estimated to be a change of just one piece of fruit per 50 customers [33]. A study conducted in University dining halls found no evidence of an intervention effect on reported fruit or vegetable intake of students [28]. One study investigated the use of recorded, morning audio-announcements promoting bean dishes in primary schools. No evidence was found that this influenced the selection of bean based dishes [31]. Although a sub-analysis of matched school pairs suggested that in the school where the announcement was played the most often, children were 2.5 times more likely to select the bean dishes than children in the comparison schools.

#### Healthier menu items

Studies which aimed to increase selection and consumption of healthier products or decrease unhealthy products provide mixed results. Two studies were conducted in the Netherlands. One found no increase in sales of low-fat soups, sandwiches, salads or snacks [33]. The other found an increase in sales of low-fat desserts (an increase from 28.6% to 44.4%) but observed no change in sales of low-fat milk, butter, cheese or meat products [32]. Similarly, a study conducted in university dining halls found a significantly lower level of reported junk food and high-fat meat intake by students in intervention halls as compared to comparison sites; however, no effects were observed for low-fat dairy products [28]. Serving of junk food and high-fat meat products decreased by 0.1 and 0.9 servings in the intervention halls, as compared to the control halls where both products increased in sales, an increase in 1.8 serving of junk food and 0.9 servings of high-fat meat per week, (*p* = 0.01 and 0.001 respectively) [28]. A study conducted in take-away restaurants found no significant effects of an information-based cue on sales for the total number of healthier items sold [27]. Two studies which reported significant effects on selection of healthier items were vending machine studies, the presence of nature posters, activity posters and posters with Giacometti sculptures (slim, elongated figures) resulted in significantly greater selection of healthier snacks as compared to when no poster or a fun fair poster was present [35]. The first study showed the percentage of healthy snacks selected was 34% with the nature poster, 28% with the activity poster and 18% with the fun fair poster as compared to 22% with the control condition. The following study reported 58% of healthy snacks were selected when posters with Giacometti figures were present as compared to 21% in the control condition [35].

#### Alcohol

One study investigated the effects of information-based cues on alcohol. This study showed sales varied between the individual bars and corresponded to bar busyness. The study found a significant increase in sales of alcohol when music was played which contained references to alcohol as compared to music which did not refer to alcohol, this was observed in all three bars within the study (F = 11.05, *P* < 0.05) [34].

## Discussion

### Principal findings

The review does not permit firm conclusions to be made for the use of information-based cues at the point of choice to change selection and consumption of food, alcohol or tobacco products, nor to reliably estimate the likely effect sizes of these interventions. The reported results, however, clearly indicate the potential beneficial effect that these interventions have, with ten out of the 13 included interventions reporting significant intervention effects for some or all targeted products. Interventions were typically successful in increasing selection of fruit and vegetables; although studies aiming to increase the selection of other healthier products and decrease selection of unhealthy products provide more mixed results. Importantly, we identified a significant gap in the literature concerning these interventions; no included studies targeted tobacco products, and only one targeted alcohol products. In characterising the evidence base, the types of information-based cues employed, as well as the intervention deigns and outcomes were highly variable across studies. Furthermore, suboptimal conduct and reporting of the included studies raises concerns over risk of bias within individual studies and the quality of the overall evidence.

### Interpretation of findings

The interventions identified within this review fall within a broader set of physical micro-environment interventions which aim to change the proximal environment to change behaviour, and which have been described in the TIPPME intervention typology [7]. More specifically we focused on interventions classed as “information” within that typology, being those that comprise cues containing any combination of words, symbols, numbers or pictures that convey information about a product or its use [7]. Despite this broad classification, the information-based cues in this review varied considerably. In order to better characterise this evidence base, with a more descriptive level of detail we have therefore, attempted to further categorise these interventions into i) Attention cues, ii) Educational cues and iii) Implicit cues. Six of the interventions are categorised as ‘Attention’ cues, where the cues attempted to draw the consumer’s attention to the target products [25, 26, 29–31, 36]. Four studies are categorised as ‘Educational cues’, in that they focused on providing educational information about the products, in this case these studies promoted products as the healthier option [27, 28, 32, 33]. Three studies are categorised as ‘Implicit cues’, acting as primes to drive selection of the product [34, 35]. In these interventions, the link between the cue and the target product is not made explicit to the potential consumer. Instead, the cue is intended to induce or influence less conscious behavioural responses via the activation of, for example, semantic relationships or associative processes [34, 35]. This basic categorisation is emergent and based on observing where interventions share common characteristics, and it primarily serves as a descriptive function. It shares similarities with the conceptual grouping used by authors of other reviews [37]. As more research is generated in this area, development of more consistent terminology or a suitably detailed classification system will enable easier grouping of interventions within further evidence synthesis, and allow the integration of more complex theoretical or conceptual ideas.

Category of cue, as outlined above, may be important in determining which cue has the greater effect on selection and consumption. With the exception of the study conducted by Achabal et al. [25], the cues which used brightly coloured images appeared to have a significant effect on increasing selection of fruit and vegetables. These cues were positive in nature and potentially acted to build on previous knowledge regarding the health benefits of fruit and vegetables. Notably, the study by Achabal et al was carried out prior to the introduction of the five a day message in the United States. It was not until 1991 that The National Cancer Institute and the Produce for Better Health Foundation created the 5 A Day for Better Health Program [38]. It is possible that when this study was conducted people were less informed regarding the health benefits of fruit and vegetables. The four studies which provided educational, health promoting cues appeared to be the least effective on changing selection and consumption [27, 28, 32, 33]. These cues may have been less effective in relation to the ‘Attention’ cues, as they generally focused on low-fat products. It has been suggested that different psychological and social processes maybe involved in reducing fat consumption, that is restricting a behaviour, compared to increasing or starting a behaviour, for example increasing fruit and vegetable intake [39].

Degree of exposure to the information-based cue may be important to their observed effect, and should be considered. The study conducted by Folta et al. which investigated the use of recorded, morning audio-announcements promoting bean dishes found no overall evidence that the announcements influenced the selection of these dishes [38]. Notably, however, a sub-group analysis of matched school pairs suggested that in the school where the announcement was played the most often, children were 2.5 times more likely to select the bean dishes than children in the comparison schools (OR 2.49, 1.74–3.53, absolute figures are not presented by the authors). The extent of exposure may also be a consideration for the other studies within this review. For example in both studies carried out by Payne and colleagues [29, 30], consumers were exposed to the intervention for the entire time they were in the supermarket. In addition, children in the study conducted by Hanks et al [26] were exposed to the vegetable characters for the entire time they were queuing for their lunch. These studies may have provided greater exposure to the intervention as compared to other studies included in the review. Indeed, Achabal et al suggest a potential explanation for no observable intervention effect in their study may have been because shoppers missed the signs due to their positioning [25].

### Implication of findings in relation to previous research

The majority of previous reviews that focus on environmental interventions within physical micro-environment have solely examined dietary interventions. This review is novel in examining the effect of information-based cues on food, alcohol and tobacco products. As such, it was able to identity a clear gap in the evidence regarding both alcohol and tobacco products. Only one study concerning alcohol was included, and we did not identify any studies which attempted to change selection or consumption of tobacco products.

Our results are consistent with previous related reviews on nutritional environmental interventions. A review of workplace interventions found more than half (59%) of included studies reported significant effects on behaviour to increase fruit and vegetable intake, increase selection of healthier options and reduce calorie intake [40]. A review of obesity related interventions in grocery stores and supermarkets found they were generally effective in stimulating purchasing and consumption of healthier foods [41]. Within both reviews, the majority of included interventions contained multiple components which the authors acknowledged precluded isolating the independent impact of included intervention components. We purposefully restricted our review to interventions comprising only information-based cues to avoid this problem.

In line with previous reviews, interventions within supermarkets [41] and vending machines [42, 43] appeared to be effective in influencing food selection. In relation to supermarkets, two of the three included studies observed significant increase in sales of fruit and vegetables [29, 30]. It has been proposed that supermarkets are effective locations for environmental interventions as people make a large percentage of unplanned purchases in store [44]. This would likely similarly apply to vending machines which are designed to cater to convenient, small scale purchasing.

### Strengths and limitations

This review is, to our knowledge novel in its focus, and describes a body of evidence that had not previously been synthesised. While intervention characteristics varied across included studies, precluding quantitative synthesis, we have both described studies in detail and attempted to further categorise interventions to enhance understanding of the existing evidence. Importantly, the review was conducted in line with the Cochrane Handbook for Systematic Reviews [20] and the PRISMA guidelines [19]. We developed an inclusive search strategy, encompassing multiple databases to capture as many relevant articles as possible, although we cannot exclude the possibility that some eligible articles were missed. The current review is limited by the suboptimal reporting of methods and outcome data of included studies, making evidence synthesis difficult, and disallowing firm conclusions about probable effects of these interventions. Future primary research studies would benefit from adhering to available guidelines for reporting standards [45, 46].

## Conclusion

Existing evidence suggests information-based cues can influence selection and consumption of food and alcohol products, although significant uncertainty remains. The current evidence base is limited both in quality and quantity, with relatively few, heterogeneous studies at unclear or high risk of bias. Additional, more rigorously conducted studies are warranted to better estimate the potential for these interventions to change selection and consumption of food, alcohol and tobacco products.

## Additional files

Additional file 1: **Table S1.** Example search strategy (MEDLINE In process& other non-indexed citations and OVID MEDLINE (R) 1946 to present). (DOCX 15 kb)

Additional file 2: **Table S2.** Cochrane risk of bias summary details. (DOCX 22 kb)

## Abbreviations

POC: Point of choice (POC)
POP: Point of purchase
TIPPME: Typology of Interventions in Proximal Physical Micro-environments
