# Supplementary material for: Information-based cues at point of choice to change selection and consumption of food, alcohol and tobacco products: a systematic review
Source: BMC Public Health. 2018 Mar 27;18:418. doi: 10.1186/s12889-018-5280-5 (PMC5872569; doi:10.1186/s12889-018-5280-5)
Supplement: Supplementary file 1 — Table S1. Example search strategy (MEDLINE In process& other non-indexed citations and OVID MEDLINE (R) 1946 to present). (DOCX 15 kb) [file 12889_2018_5280_MOESM1_ESM.docx]

| **MEDLINE In process& other non-indexed citations and OVID MEDLINE (R) 1946 to present** |
| --- |
| 1 exp Beverages/ 119090  2 *Alcohol Drinking/ 40657  3 *Drinking/ 4794  4 (drink$ or drunk$ or alcohol$ or beverage$1 or beer$1 or lager$1 or wine$1 or cider$1 or alcopop$1 or alco-pop$1 or spirit$1 or spirits or liquor$1 or liquer$1 or liqueur$1 or whisky or whiskey or whiskies or whiskeys or schnapps or brandy or brandies or gin or gins or rum or rums or tequila$1 or vodka$1 or cocktail$1 or perry$1 or ale$1 or sherry$1 or vermouth$1 or sangria$1 or champagne$1 or prosecco$1).ti,ab. 427243  5 *Tobacco/ 18203  6 *Smoking/ 79006  7 (cigar$ or smok$ or tobacco$ or e-cig$ or pipe$).ti,ab. 365388  8 exp Food/ 1171774  9 *Food Intake/ 19026  10 *Food Habits/ 15349  11 *Food Preferences/ 7041  12 *Eating/ 19026  13 *Food Dispensers, Automatic/ 207  14 "Nutrition education".ti,ab. 3681  15 (grocer* adj3 (shop* or store*)).ti,ab. 1335  16 (food$ or eat or eats or eaten or eating or consume or ate or low-fat or meal$ or dessert$ or snack$ or drink$ or beverage$ or pudding* or confection* or candy or sweets or vegetable* or fruit*).ti,ab. 669858  17 1 or 2 or 3 or 4 or 5 or 6 or 7 or 8 or 9 or 10 or 11 or 12 or 13 or 14 or 15 or 16  2244888  18 "point of purchase".mp. 245  19 "point of choice".mp. 91  20 "point of sale".mp. 376  21 (P-O-P or P-O-C or P-O-S).mp. 249  22 "information cue$".mp. 56  23 "environmental cue$".mp. 5705  24 ((cue$ or information$ or menu$ or intervention$ or promotion$ or market$ or nutrient$ or nutrition$ or food$ or alcohol$ or tobacco$) adj7 (sign$ or placard$ or banner$ or poster$ or board$ or flyer$ or label$ or symbol$ or logo$ or display$ or arrow$)).ti,ab. 146615  25 (market$ adj7 intervention$).ti,ab. 750  26 18 or 19 or 20 or 21 or 22 or 23 or 24 or 25  153032  27 (shop$ or store$ or supermarket$ or market$ or hypermarket$ or outlet$ or grocer$ or retailer$ or stall$ or restaurant$ or cafe$ or bar$ or canteen$ or cafeteria$ or dinner hall$ or dining area$ or dining room$ or refector$ or eatery or mess or buffet or bistro$ or eating place$).ti,ab. 781662  28 exp restaurants/ 3652  29 exp commerce/ 59585  30 27 or 28 or 29  824690  31 17 and 26 and 30  6731  32 exp animals/ not humans/ 4669475  33 (rat or rats or mouse or mice or murine or rodent or rodents or hamster or hamsters or pig or pigs or porcine or rabbit or rabbits or animal or animals or dog or dogs or cat or cats or cow or cows or bovine or sheep or ovine or monkey or monkeys).ti,ab. 4047420  34 32 or 33  5846223  35 31 not 34 5602  36 (editorial or case reports or letter or comment).pt. 3283923  37 35 not 36  **5557** |
