# Supplementary material for: Information-based cues at point of choice to change selection and consumption of food, alcohol and tobacco products: a systematic review
Source: BMC Public Health. 2018 Mar 27;18:418. doi: 10.1186/s12889-018-5280-5 (PMC5872569; doi:10.1186/s12889-018-5280-5)
Supplement: Supplementary file 2 — Table S2. Cochrane risk of bias summary details. (DOCX 22 kb) [file 12889_2018_5280_MOESM2_ESM.docx]

|  | **Random sequence generation** | **Allocation concealment** | **Blinding of participants and personnel** | **Blinding of outcome assessment** | **Incomplete outcome data** | **Selective reporting** | **Other sources of bias** |
| --- | --- | --- | --- | --- | --- | --- | --- |
| **Achabal 1987** | Unclear  “a random assignment was used”  “assignment was made in a totally random order” | Unclear  Not described | Low  No blinding possible for participants, but unaware study is in operation due to nature of the study. Highly unlikely that key study personnel were blinded, but the review authors judge that the outcome is not likely to be influenced by lack of blinding of key study personnel. | Low  Blinding not stated; however, data is collected via objective means (sales reports), thus review authors judge outcome is not likely to be influenced. | Low  No missing outcome data | Unclear  Mean and N values not stated, but full analysis of covariance results presented. | Unclear  Data for the individual intervention arms not clearly separated in analysis, making interpretation unclear |
| **Payne 2015** | High  No random allocation | High  No random allocation | Low  No blinding possible for participants, but unaware study is in operation due to nature of the study. Highly unlikely that key study personnel were blinded, but the review authors judge that the outcome is not likely to be influenced by lack of blinding of key study personnel. | Low  Blinding not stated; however, data is collected via objective means (sales reports), thus review authors judge outcome is not likely to be influenced. | Low  No missing outcome data | High  No mean data or n values. Only partial reporting of overall results | Unclear  Overall study is poorly described and reported, making interpretation difficult |
| **Payne 2016** | High  No random allocation | High  No random allocation | Low  No blinding possible for participants, but unaware study is in operation due to nature of the study. Highly unlikely that key study personnel were blinded, but the review authors judge that the outcome is not likely to be influenced by lack of blinding of key study personnel. | Low  Blinding not stated; however, data is collected via objective means (computer tape of sales), thus review authors judge outcome is not likely to be influenced. | Low  No missing outcome data | High  No mean data or n values. Only partial reporting of overall results | High  Limited detail provided in the methods, results are not completely presented, review authors judge overall risk of bias is high. |
| **Hanks 2016** | Unclear  “schools were randomly assigned” | Unclear  No reference to allocation concealment | Low  No blinding possible for participants, but unaware study is in operation due to nature of the study. Highly unlikely that key study personnel were blinded, but the review authors judge that the outcome is not likely to be influenced by lack of blinding of key study personnel. | High  Count data was by researchers who would be able to see the intervention whilst recording data;  “Researches used a right clicker to tally male students and a female clicker to tally female students visiting the salad bar” | Low  No missing outcome data | High  Most data presented in graphical format.  Full data on main outcomes not reported for each intervention arm. | Unclear  Data collection methods potentially unreliable. |
| **McClain 2012** | Unclear  Study reported as Cluster-randomised, but no reference to randomisation process in methods | Unclear  Not described | Low  No blinding possible for participants, but unaware study is in operation due to nature of the study. Highly unlikely that key study personnel were blinded, but the review authors judge that the outcome is not likely to be influenced by lack of blinding of key study personnel. | High  Surveyors approached students in both control and intervention halls.  FFQ data completed by students who were aware of the intervention in the dining hall. | Unclear  No reference if missing data were equal across groups etc. | Low  Expected outcome variables presented. | Unclear  Unclear if the students involved with the study design were those completing the FFQ |
| **Steenhuis 2004** | Unclear  “Cafeterias were randomly assigned” | Unclear  Not described | Low  No blinding possible for participants, but unaware study is in operation due to nature of the study. Highly unlikely that key study personnel were blinded, but the review authors judge that the outcome is not likely to be influenced by lack of blinding of key study personnel. | High  Unclear how the cohort of visitors who completed the questionnaires were selected.  Low  Sales data collected is objective; however, methods state “some targeted product categories, were collected”  Overall: Unclear | Unclear  No reference if missing data were equal across groups etc.  Low  For sales data  Overall: Unclear | Low  Expected outcome variables presented. | Unclear  Intervention not fully detailed in the methods. Unclear how participants were selected to complete questionnaire, indicating risk of selection bias. |
| **Morizet 2012** | Unclear  The temporal order in which the children were presented with both types of vegetables was randomized, as was their assignment to each condition” | Unclear  Not described | Unclear  No blinding for participants, unclear if children were aware they were in an intervention regarding the use of information-based-cues as this is not reported in the methods. | High  Outcome measured by a camera, which will require researchers to interpret the images | Low  No missing outcome data | Unclear  Most data presented in graphical format without numbers presented. | High  Independence assumption is likely to be broken if children observe others picking a certain plate (this also may conflate the main effect of labelling vs. imitation of peers). Analysis should take into account that children are being randomised by cluster. |
| **Lee-Kwan 2015** | High  “four carry outs were randomly selected per stratum, four comparison carry outs were matched”  Does not appear to be a randomised | Unclear  No description provided | Low  No blinding possible for participants, but unaware study is in operation due to nature of the study. Highly unlikely that key study personnel were blinded, but the review authors judge that the outcome is not likely to be influenced by lack of blinding of key study personnel. | Low  Blinding not stated; however, data is collected via objective means (weekly sales receipts), thus review authors judge outcome is not likely to be influenced. | Low  No missing outcome data | Unclear  Unclear presentation of the different phases of the intervention.  Do not clearly present intervention vs. comparison | Unclear  New menu items were introduced; this may have influenced selection by regular customers. Overall data poorly presented |
| **Stockli 2016 (study 1)**  **& Stockli 2016 a (study 2)** | Unclear  “the four poster conditions were permutated and counterbalanced across the three test locations” | Unclear  Not described | Low  No blinding possible for participants, but unaware study is in operation due to nature of the study. Highly unlikely that key study personnel were blinded, but the review authors judge that the outcome is not likely to be influenced by lack of blinding of key study personnel. | Low  Blinding not stated; however, data is collected via objective means (daily inventory snack sales), thus review authors judge outcome is not likely to be influenced. | High  Do not report data from other products, this could shift sales. | High  Percentages presented in graphical format. Not all numbers, or analysis provided | High  With randomisation by cluster the use of chi-squared tests is inappropriate  Review authors believe the use of chi-squared test is inappropriate for the study design |
| **Engels 2011** | Unclear  “all evenings were randomly assigned” | Unclear  Not described | Low  No blinding possible for participants, but unaware study is in operation due to nature of the study. Highly unlikely that key study personnel were blinded, but the review authors judge that the outcome is not likely to be influenced by lack of blinding of key study personnel. | Low  Blinding not stated; however, data is collected via objective means (evening turnover), thus review authors judge outcome is not likely to be influenced. | Low  No missing outcome data | High  No mean data on evenings in different bars.  Excluded some evenings as stated they were not representative, would have been better to do some additional analysis with and without, unclear if this was for all bars. | High  Data potentially not reported fully  Turnover is not a direct measure of alcohol consumed  Other differences in songs could have had an influence  Busyness measured subjectively by bar staff |
| **Folta 2006** | Unclear  “one school from each pair was randomly selected | Unclear  Not stated | Low  No blinding possible for participants, but unaware study is in operation due to nature of the study. Highly unlikely that key study personnel were blinded, but the review authors judge that the outcome is not likely to be influenced by lack of blinding of key study personnel. | High  Choice of bean recorded by observers. Observers also spoke to the children to ask their grade. | Low  No missing outcome data | Unclear  Do not provide mean data, but OR and CI presented for primary outcomes | High  Data collection methods  Discussion based on investigative analysis of data, not the primary outcome finding. |
| **Vyth 2011** | Unclear  No statement of how randomisation occurred.  But state “randomisation was stratified by company size” | Unclear  worksites were “blindly allocated to either intervention or control”  Sites had “assignment codes” | Low  No blinding possible for participants, but unaware study is in operation due to nature of the study. Highly unlikely that key study personnel were blinded, but the review authors judge that the outcome is not likely to be influenced by lack of blinding of key study personnel. | Low  Blinding not stated; however, data is collected via objective means (daily sales data), thus review authors judge outcome is not likely to be influenced. | Low  Missing data imputed; “ missing data were accounted for with the multiple imputation method for missing data” | Unclear  No mean or number values presented. Yet OR and 95%CI for primary outcomes presented. | Unclear  Results section brief with little information. |
